# Deformation Analysis of the Glass Preform in the Progress of Precision Glass Molding for Fabricating Chalcogenide Glass Diffractive Optics with the Finite Element Method

**DOI:** 10.3390/mi12121543

**Published:** 2021-12-11

**Authors:** Yue Liu, Yintian Xing, Hang Fu, Chuang Li, Chao Yang, Bo Cao, Changxi Xue

**Affiliations:** 1 School of Optoelectronic Engineering, Changchun University of Science and Technology, Changchun 130022, China; liuyue_changchun@sina.com (Y.L.); xingyintian@163.com (Y.X.); fuhang19020182@163.com (H.F.); lichuang_2012@sina.com (C.L.); yangchaoby@sina.com (C.Y.); 2 Key Laboratory of Advanced Optical System Design and Manufacturing Technology of the Universities of Jilin Province, Changchun University of Science and Technology, Changchun 130022, China; 3 GRINM Guojing Advanced Materials Co., Ltd., Langfang 065001, China; caobo@guojing-tech.com

**Keywords:** precision glass molding, finite element method, glass deformation

## Abstract

Precision glass molding (PGM) technology is a cost-efficient process for the production of micro/nanostructured glass components with complex surface geometries. The stress distribution, surface profile, and reduced refractive index of the molded lens are based on the lens being fully formed. The process of the deformation of the glass preform is rarely discussed, especially in the case of multi-machining parameters in the experiment. The finite element method (FEM) was adopted to analyze the glass preform deformation. Due to the phenomenon of incomplete deformation of the glass preforms in the experiments, two groups of finite element simulations with different boundary conditions were carried out with MSC.Marc software, to reveal the relationship between the deformation progress and the parameters settings. Based on the simulation results, a glass preform deformation model was established. The error between the model result and the simulation result was less than 0.16. The establishment method of the glass preform deformation model and the established model can be used as a reference in efficiently optimizing PGM processing parameters when the designed lens has two different base radii of curvature.

## 1. Introduction

Glass optical components are widely used in the camera, computer, laser projection, biological, and consumer electronics fields because of their optical performance, mechanical stability, thermal stability, and high chemical resistance [1,2,3,4]. The hybrid aspherical diffractive singlet achromat design can be used to reduce chromatic aberration in compact optical systems. The applications are almost in the areas of bifocal contact lenses, beam shapers, and barcode reader diffusers [5].

Precision glass molding (PGM) technology is a cost-efficient process for the production of micro/nanostructured glass components with complex surface geometries [6,7,8]. It is a technology that deforms glass preform into the desired shape by use of molds at a high temperature in an inert or vacuum environment [3,9,10,11].

Among optical glass, chalcogenide glasses (ChGs) are a group of materials that contain one or more of the chalcogen elements (S, Se, and Te) and group IIIA-VA elements (Ge, Ga, As, and Sb). ChG has a wide optical window that can be extended from the visible to far-infrared spectrum [12]. Compared to Ge or ZnSe, ChGs are amorphous and can be fabricated to the lenses with PGM, which provides a significant advantage over diamond turning due to its high-volume manufacture and lower cost. The densities and *dn*/*dT*s of ChGs are lower than those of Ge, thus having advantages in weight and athermalization. In addition, ChGs have low dispersion, low cost, high optical uniformity, and excellent properties in achromatism [9,13,14,15,16]. However, ChGs are soft optical materials. To withstand abrasion during normal operation and improve transmittance, DLC (diamond like carbon) is generally applied to ChGs lenses [12,17]. Among the ChGs, Ge_x_Se_1−x_ is a binary covalent glass that has been investigated extensively for its outstanding transparent property [18]. The low production cost prompt the ChG glass lens to be applied more in infrared optical systems [19]. However, fabricating high quality diffractive glass optics in micrometer size with PGM is still challenging, preventing it from being widely adopted in commercial applications [2,13,15].

Around the molding temperature, ChG is in a viscoelastic state [10]. The finite element method has become an efficient method for PGM for predicting the molding force, profile deviation, residual stress of the lens, and birefringence distribution, and for designing the appropriate processing parameters for industrial purposes [3,11,20,21]. Zhou et al. analyzed the refractive index changes of ChG As_2_S_3_ lens with the cooling rates of finite element method (FEM) to verify and modify the calculated stress relaxation function [22]. They also got the results of curve deviation, temperature gradient, and residual stress of a plano-concave molded glass lens, with sets of variations in effective diameter, sag ratio, and center thickness ratio through thermal-displacement coupled finite element analysis [23]. Zhang et al. simulated the index shift of the refractive index of the molded ChG As_40_Se_50_ S_10_ lens and propose that the optical design should take the post-molding refractive index into consideration [24]. The processing parameters are optimized by the finite element method to validate the feasibility of mold surface compensation [2,15]. Zhou et al. optimized the processing parameters to make a balance between the molded lens stress and the molding temperature with FEM when molding the Fresnel lens [25]. FEM also can be used to investigate the formation and propagation of the cracks of the molded lens [26].

The deformation of the glass preform was determined by the relative position of the upper and lower molds to a great extent. In a PGM experiment, the displacement of the mold was controlled by several setting parameters, including pressing force, pressing speed, press slope, and mold temperature. However, the effects of these parameters on the glass preform deformation are still scarcely addressed, especially for cases of molding with fixed die machines. The PGM machine usually has a unique execution program for a specific set of processing parameters, which is not known to the users. The unknown relationship between the designed parameters and the actual parameters will be a barrier for explaining the experimental phenomena and optimizing the processing parameters in the research and development cycle. Therefore, the interaction influence of multiple machining parameters on the glass preform deformations needs to be addressed.

The remainder of this paper is organized as follows. Section 2 presents the monitoring data of the pressing force and displacement of the lower shaft under different processing parameters when fabricating ChG diffractive-refractive hybrid lenses on a fixed die PGM machine. Section 3 states the strain-time relationship of the glass during the creep process. Two groups of finite element simulations when the displacement or the pressure separately was the boundary condition were carried out, and the simulation results were discussed to establish the glass preform model for molding the ChG diffractive optics in Section 4. Finally, the study is summarized in Section 5. The established glass preform deformation model explains the phenomenon of the deformation rate decreasing while the pressing force increased and reveals the effect of the multiple machining parameters on the glass preform deformations. This study will gain attention from other researchers who work on designing deformation rates of the two surfaces of the glass preform and the geometry of the glass preform, and optimizing the processing parameters.

## 2. Materials and Methods

**Figure 1:**
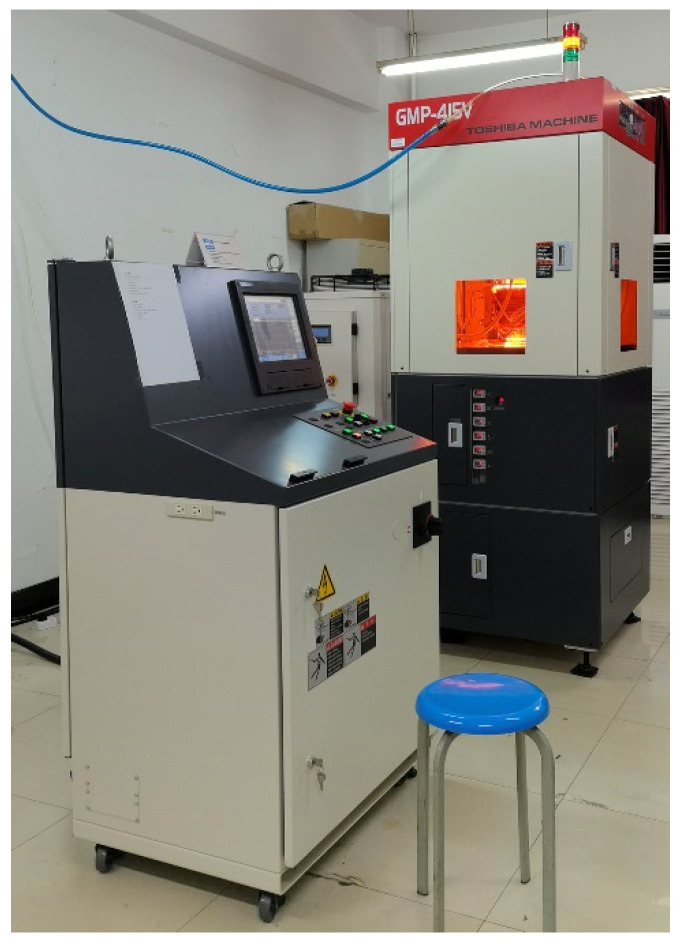
The photograph of the precision glass mold press machine (Model GMP-415V) from Toshiba Machine Co., Ltd.

Chalcogenide glass diffractive-refractive hybrid lenses are molded on a Toshiba precision glass molding machine (Model No. GMP-415V, Toshiba Machine Co., Ltd., Numazu, Japan), as shown in Figure 1. The diameter and thickness of the designed lens are 22 mm and 4 mm, respectively. The aspherical surface and the hybrid aspherical diffractive surface are respectively described with Equations (1) and (2).

(1) $$z_{1}=\frac{c_{1}r_{1}^{2}}{1+\sqrt{1-\left(1+k_{1}\right)c_{1}^{2}r_{1}^{2}}}+A_{2}r_{1}^{2}+A_{4}r_{1}^{4}+A_{6}r_{1}^{6}+A_{8}r_{1}^{8}+A_{10}r_{1}^{10},$$

(2) $$z_{2}=\frac{c_{2}\cdot r_{2}^{2}}{1+\sqrt{1-\left(1+k_{2}\right)c_{2}^{2}r_{2}^{2}}}+B_{2}r_{2}^{2}+B_{4}r_{2}^{4}+B_{6}r_{2}^{6}+B_{8}r_{2}^{8}+B_{10}r_{2}^{10}+\frac{1}{1-n}\left(C_{1}r_{2}^{2}+C_{2}r_{2}^{4}\right).$$

In Equation (1), *c*_1_ is the curvature at the center of the profile, *k*_1_ is the conic constant, *r*_1_ is the radius of the lens, and *A*_2_, *A*_4_, *A*_6_, *A*_8_, and *A*_10_ are the even order coefficients. In Equation (2), *c*_2_ is the curvature at the center of the profile, *k*_2_ is the conic constant, *r*_2_ is the radius of the lens, *n* is the coefficient of refraction of the lens, and *B*_2_, *B*_4_, *B*_6_, *B*_8_, and *B*_10_ are the even order coefficients of the aspheric base. The material of the lens is Schott grade IRG26. The coefficients of the designed lens are listed in Table 1.

**Figure 2:**
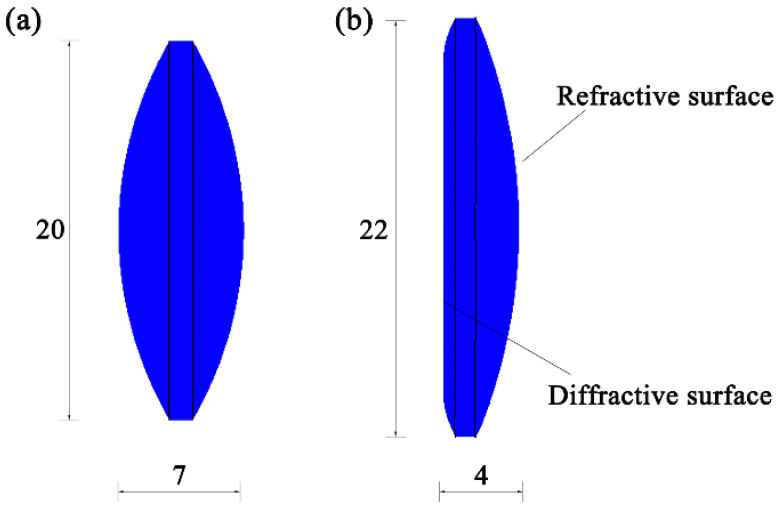
Schematic diagrams of the (**a**) glass preform and (**b**) designed lens.

The glass preform is a biconvex spherical lens with a curve radius of 20 mm, a thickness of 7 mm, and a diameter of 20 mm. The diagram of the designed lens and the glass preform is shown in Figure 2.

**Figure 3:**
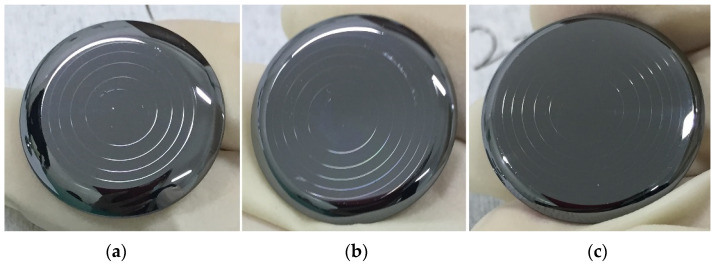

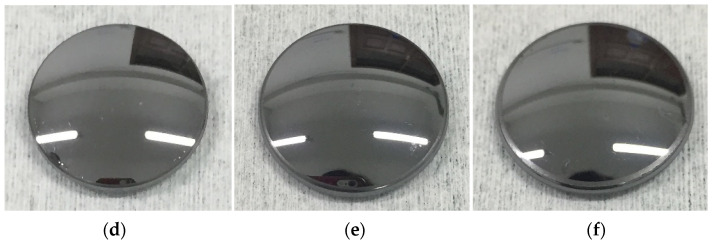
Photographs of diffractive surfaces and the aspherical surfaces of the molded lens: (**a**,**d**) the lens surfaces in the No. 1 experiment; (**b**,**e**) the lens surfaces in the No. 2 experiment; (**c**,**f**) the lens surfaces in the No. 3 experiment.

Rapidly solidified aluminum RSA-905 was selected as the mold core material. Three PGM experiments were carried out with the processing parameters listed in Table 2. The photographs of the molded lens are shown in Figure 3. The difference among the molded diffractive surfaces was obvious, while the difference among the molded aspherical surfaces was small. 

**Figure 4:**
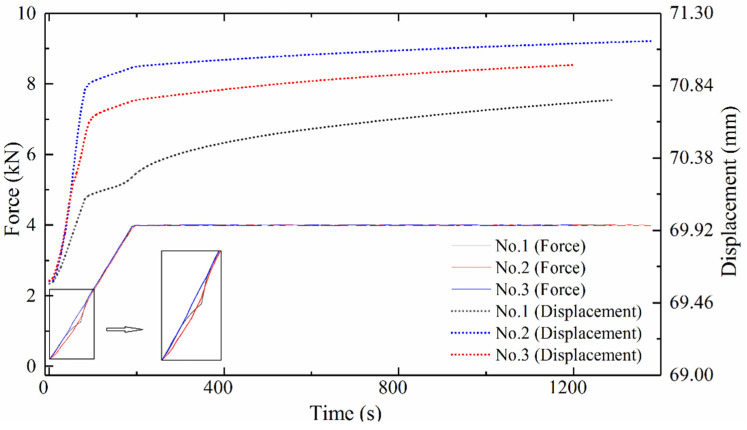
The waveforms of the force and displacement of the lower mold.

A compact flash card can be inserted into the card slot of the PGM machine to store the data detected during automatic operation. The sampled data is saved in a file at each cycle. Sampling was performed from the start to the end of a cycle. The sampling period was set as 9000 s. One thousand data items were collected within the sampling period. That is, the sampling cycle is sampling period/1000 items, which is nine seconds. The waveforms of the force and displacement of the lower mold since the forming stage began are shown in Figure 4. 

The deformation of the glass preform is related to the relative position of the upper and lower molds. The upper mold was installed on the fixed upper axis of the molding system, while the lower molds were installed on the mobile lower axis. Therefore, there is a need to describe the displacement of the lower axis with time. The displacement data are processed with regression analysis with IBM SPSS Statistics software (25, SPSS Inc., Chicago, IL, USA). When the time is *t*, the displacement is *y_i_* for the *i*th experiment. Based on the results, the line types of the curve consists of linear, conic, and cubic curves. The three kinds of curves are expressed as Equations (3)–(5).

(3) $$y=a_{1}+a_{2}\times t,$$

(4) $$y=b_{1}+b_{2}\times t+b_{3}\times t^{2},$$

(5) $$y=d_{1}+d_{2}\times t+d_{3}\times t^{2}+d_{4}\times t^{3}.$$

The fitting parameters are listed in Table 3 when R-squared is bigger than 0.98. Whichever line type was chosen, the slope of the displacement of No. 3 was either 200 times of the No. 1, or 10 times of No. 2, before the force reached the setting values. Therefore, the actual press speed may be a function depending on the set values of the pressing force, speed rate, press slope, and molding temperature. Then, there are two phenomena that need to be explained. The first one is why the pressing force was still rising, when the deformation rate decreased from 81 s to 200 s. The second one is why the relationships of the deformation velocities among the three simulations were not the same as the relationships among the processing parameters.

## 3. Viscoelasticity of Glass 

When applying specified stress on glass at the molding temperature, strain (*ε*(*t*)) goes on increasing with time (*t*), which is called creep. In creep, the deformation of glass is a superposition of instantaneous elastic deformation, delayed elastic deformation, and viscous flow, which can be described by viscoelastic mechanical models. When applying a constant stress *σ*_0_ at *t* = 0, the strain–time relationship can be obtained by Equations (6) and (7) according to Kelvin model [27].

(6) $$\varepsilon \left(t\right)=\frac{\sigma_{0}}{E}\left(1-e^{-\frac{t}{\tau }}\right),$$

(7) $$\tau =\frac{\eta }{E},$$

*E* and *η* are elastic modulus and viscosities of the viscoelastic material, respectively. *τ* is defined as the term “retardation time” to describe the creep rate in the viscoelastic deformation. 

## 4. Deformation Analysis with FEM and Discussion

The deformation of the glass includes two parts, which are the deformation of the upper half part and the lower half part of the glass preform. The respective deformation speeds of the upper half and the lower part are not known from the monitoring values in the experiment. FEM could be helpful for analyzing the deformation. The boundary condition could be displacement control and the element surface pressure control. The simulation model is firstly established, and then two groups of simulations with the two kinds of boundary conditions are carried out. 

### 4.1. Simulation Model

To gain more information about the simulation result of the molded lens, three-dimensional simulation was carried out. If the geometries of the molds were the same as the actual molds, the computation workload would be too large. Thus, the profile of the mold core was the same as the design, but the height of the mold core was smaller than the actual mold core. As the simulation in this paper only included the molding stage of the PGM process, which does not involve heat transfer, the simplified geometry model would be acceptable.

**Figure 5:**
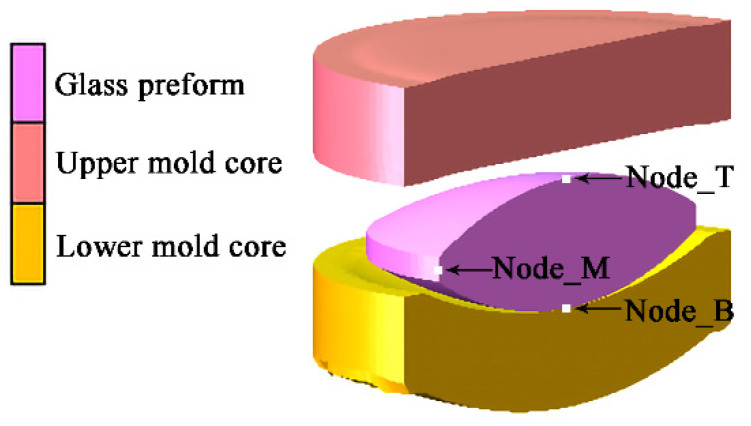
The diagram of the established three-dimensional geometry model of the molds and glass preform.

The three-dimensional geometric model is established in the finite element analysis software MSC.Marc (2016.0.0, MSC Software Corp., Newport Beach, CA, USA), as shown in Figure 5. There were three contact bodies including the upper and lower molds and the glass preform. The geometries of the upper and lower molds were artificially and subjectively divided into 20,335 and 20,638 elements, which include pentahedrons and hexahedrons. The geometry of the glass preform was divided into 66,206 elements, which were tetrahedrons. The use of these finite elements can meet the requirements of geometric model construction.

**Figure 6:**
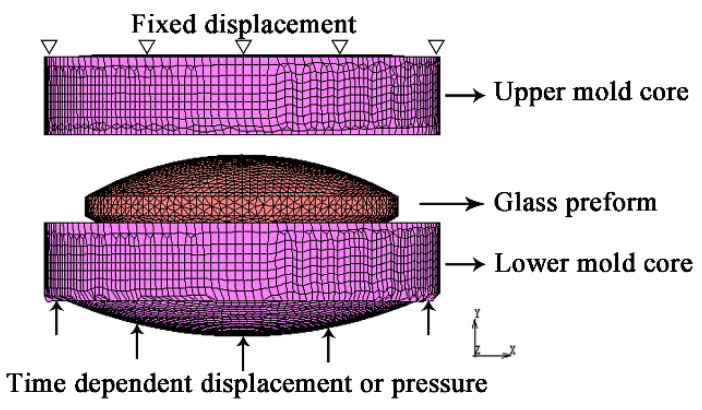
The diagram of established finite element model with boundary conditions.

The PGM machine instruction book shows that the pressing force that is detected by the load cell is displayed in real-time. However, when the actual pressing forces that were applied to the glass were the same, the theoretical deformations should be the same, which were different from the actual deformations shown in Figure 4. Thus, the actual pressing force should be a function dependent on other relative setting processing parameters. Therefore, the recorded forces should not be the boundary conditions. The pressures applied to the mold core in the simulations were separately designed for qualitative analysis. As the values of the boundary conditions were not the processing parameters of the experiments, the viscoelastic parameters were used for calculating the simulations to describe the glass deformation over time. The use of the viscoelastic parameters of different glasses at the temperatures which make the glasses have a similar viscosity was accepted in this paper. The viscoelastic parameters of glass D-ZK3L at 550 °C, which can be a molding temperature, were used for the finite element simulations. The viscoelastic properties of glass D-ZK3L, other thermal properties of glass IRG206, and mechanical properties of the mold were listed in Table 4 [28,29,30]. The diagram of the established finite element model with boundary conditions is shown in Figure 6. The boundary conditions were applied to the end faces of the mold cores.

According to the simulation results, the displacement in y direction of any node could be obtained. The displacement data of the three nodes were extracted: the center node of the top surface of the glass preform (Node_T in Figure 5), the center node of the bottom surface of the glass preform (Node_B in Figure 5), and the edge intermediate node of the glass preform (Node_M in Figure 5). Therefore, the total deformation of the glass preform can be calculated by subtracting the displacement of Node_B from the displacement of Node_T. The deformation of the upper part of the glass preform can be calculated by subtracting the displacement of Node_M from the displacement of Node_T. Therefore, the deformation of the lower part of the glass preform can be calculated by subtracting the total deformation from the deformation of the upper part of the glass preform. 

### 4.2. Simulation with the Boundary Condition of Displacement Control

To analyze the different deformation speeds of the upper and lower parts of the glass when the total deformation is linear, the displacement of the lower mold is the boundary condition. A group of simulations were carried out. The deformations of the two half parts of the glass preform were analyzed. 

**Figure 7:**
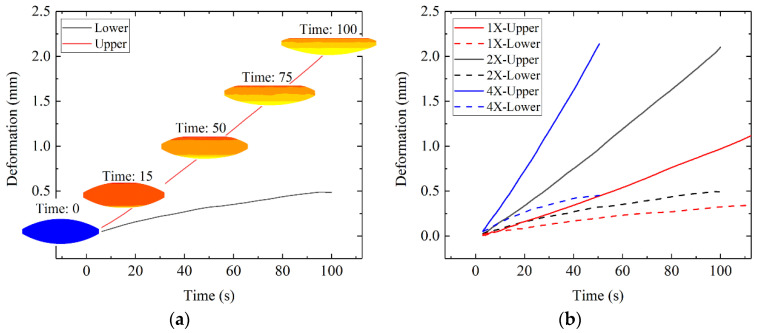
(**a**) The deformation diagram of the glass preform in the 2X experiment; (**b**) The deformation of the glass preforms in the three experiments.

A table that showed the data on displacement dependent with time was established in the software and was quoted in the process of the calculation in each simulation. The displacements of the mold were set as 0.013, 0.026, and 0.052 mm/s in the three simulations. These simulations were named 1X, 2X, and 3X. The deformation amounts of the upper half part and the lower half part of the glass and the shape of glass in the 2X experiment are shown in Figure 7a. The deformation amounts of the three simulations are plotted as three group curves as shown in Figure 7b. 

The three group curves also were processed through regression analysis with IBM SPSS Statistics software. The fitting parameters are listed in Table 5 when R-squared is bigger than 0.95. The line types of the curves representing the deformation of the upper and lower half part of the glass preform were highly linear. The slopes of the curves represent that the deformations of the upper and lower half part of the glass preform in the three simulations nearly had the same multiple relationships of the three boundary conditions. When the pressure applied to the glass was enough, the deformation velocity of the upper half part and the lower half part should be constant. The sum of the two constants should be the slope of the displacement boundary condition. However, the specific value should be related to the viscosity of the glass and the curvature of the molded glass surface.

The displacement of the lower axis is highly linear in the first 81 s of the forming stage in the PGM experiments, according to Section 4.2. Therefore, the deformation of the upper half part and the lower half part of the glass was linear. For the No. 1 experiment, the deformation velocity of the upper part of the glass was assumed as *V_α_*_1_, and the deformation velocity of the lower part of the glass was assumed as *V_α_*_2_. 

The PGM machine instruction book pointed that the pressing force that was detected by the load cell was displayed in real-time. However, when the actual press forces that were applied to the glass were the same, the deformation should be the same. Thus, the actual press force is a function dependent on the time (*t*), and the actual press slope of the No. 1 experiment is assumed to be *S*_1_. When a force is applied to the glass preform with the spherical surface with a curve of 20 mm, the varying contact surface will lead to varying pressure.

**Figure 8:**
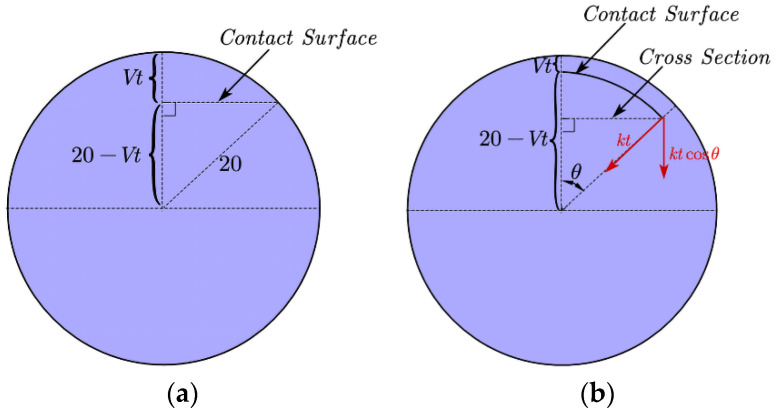
(**a**)The diagram of the upper contact surface. (**b**) The diagram of the lower contact surface.

In the progress of precision glass molding, the diffractive surface is upward and the aspherical surface is downward. The upper contact surface area, *A*(*t*), of the glass preform and the mold core can be calculated by the geometry relationship as shown in Figure 8a and can be expressed by Equation (8). The rate of change of contact area is assumed to be *a*(*t*), which can be expressed by Equation (9) when *V_α_*_1_ is smaller than 0.02 mm/s. The actual pressure *σ_α_*_1_(*t*) applied to the upper surface of the glass can be expressed as Equation (10).

The projection radius, *R_p_*(*t*), of the lower contact surface shown in Figure 8b can be expressed by Equation (11). The normal force, *F_n_*(*t*), applied to the lower contact surface can be expressed by Equation (12). The normal pressure applied to the lower surface is *σ_α_*_2_(*t*), expressed as Equation (13).

(8) $$A\left(t\right)=\pi \left[20^{2}-{\left(20-V_{\alpha 1}t\right)}^{2}\right],$$

(9) $$a\left(t\right)=\frac{dA\left(t\right)}{dt}=2\pi \left(20V_{\alpha 1}-V_{\alpha 1}^{2}t\right)≃40\pi V_{\alpha 1},$$

(10) $$\sigma_{\alpha 1}\left(t\right)=\frac{S_{1}t}{40\pi V_{\alpha 1}t}=\frac{S_{1}}{40\pi V_{\alpha 1}},$$

(11) $$R_{p}\left(t\right)=\left(20-V_{\alpha 2}t\right)\sin\left(\frac{9}{2}V_{\alpha 2}t\right),$$

(12) $$F_{n}\left(t\right)=\frac{2\int_{0}^{\frac{9}{2}V_{\alpha 2}t}S_{1}t\cos\theta d\theta }{180}=\frac{S_{1}t\sin\left(\frac{9}{2}V_{\alpha 2}t\right)}{90},$$

(13) $$\sigma_{\alpha 2}\left(t\right)=\frac{S_{1}t}{90\pi {\left(20-V_{\alpha 2}t\right)}^{2}\sin(\frac{9}{2}V_{\alpha 2}t)}.$$

**Figure 9:**
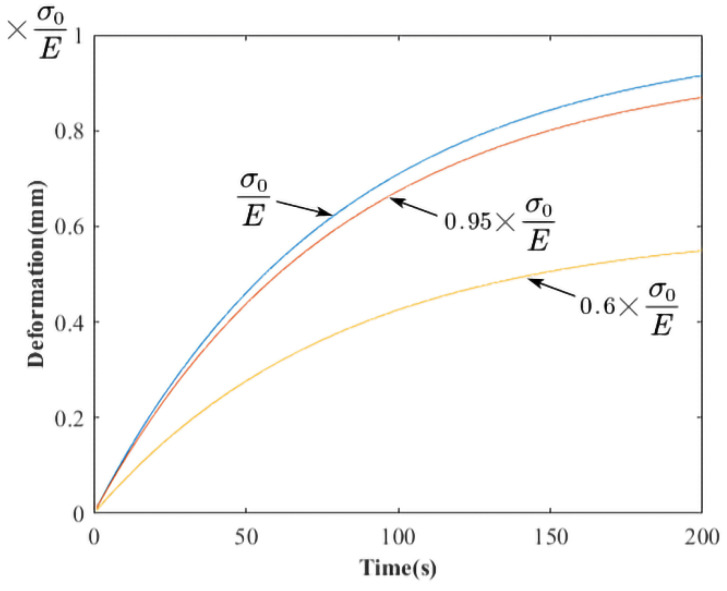
The creep curves described by Kelvin model.

The strain, *ε_α_*_1_(*t*), of the upper surface can be concluded as Equation (14), according to Kelvin model. The strain of the lower surface is much smaller than the upper surface from Figure 7b.

(14) $$\varepsilon_{\alpha 1}\left(t\right)=\frac{S_{1}}{40\pi V_{\alpha 1}E}\left(1-e^{-\frac{t}{\tau }}\right)=\alpha \left(1-e^{-\frac{t}{\tau }}\right),$$

where *α* is a calculated constant. To know the property of the curve, like in Equation (6), when *τ* is assumed to be 81 s, the curves when the parameters are *σ*_0_/*E*, 0.95 × *σ*_0_/*E*, 0.6 × *σ*_0_/*E* are plotted as in Figure 9. It was seen that the strain was highly linear from 0 s to 81 s, while the strain speed obviously decreased after 81 s. Therefore, the first phenomenon in the problem description part can be explained. The varying press force and the varying contact area led to a constant pressure before the 200 s mark in the three experiments. The strain-time curve for the viscoelasticity under constant pressure has similar properties of the curves in Figure 9.

### 4.3. Simulation with the Boundary Condition of Pressure Control

**Figure 10:**
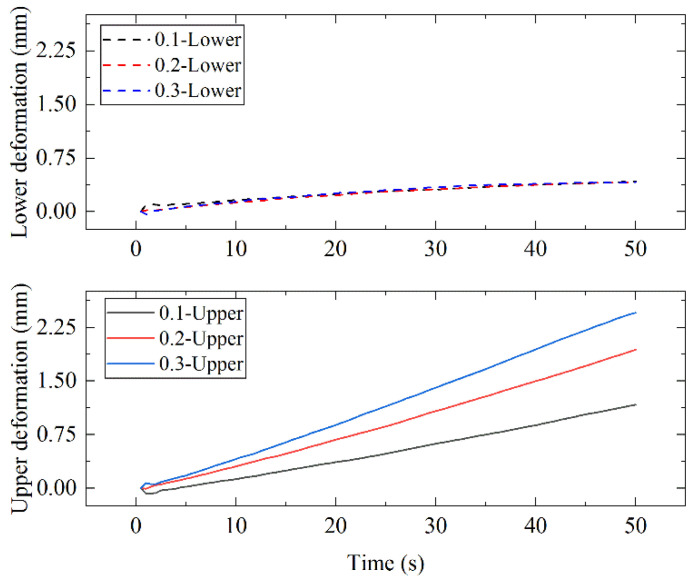
The deformation of the glass preform in the three simulations.

To obtain the deformation degree when the pressing force was different, a group of simulations were carried out. As the area of the bottom surface of the lower mold core was constant, the boundary condition in this section was the element surface distribution pressure. The pressure values that were applied to the element surface varied dependent on the time, *t*. The pressures in the No. 1, No. 2, and No. 3 simulations were 0.1 *t* MPa, 0.2 *t* MPa, and 0.3 *t* MPa, respectively. The personal computer parameters and the simulation operation times were listed in Table 6. Since it was a three-dimensional simulation, it took nearly two hours to calculate each simulation. The deformations of the glass preforms in the three simulations are plotted as curves, as shown in Figure 10. The deformations of the lower half part of the glass preforms were nearly the same, but there were many big differences in the deformation of the upper half part of the glass preforms. The deformations of the upper half part of the glass preforms were nearly linear. The deformation rates were about 0.025, 0.04, and 0.051 mm/s in the three simulations, according to IBM SPSS Statistics software. The relationships of the deformation rates were not the same as those of the applied pressures in the simulations.

The actual press force should be a function with the setting press speed. According to Section 4.2, the actual press slope of the No. 1 experiment was assumed to be *S*_1_. Therefore, the actual press slopes of the No. 2 and No. 3 experiments were assumed to be *S*_2_ and *S*_3_. Since the upper deformation was similarly linear, the strain calculation was similar to that in Section 4.2. Therefore, the rate of the strain *V_α_*_1_(*t*) of the upper half part of the glass in the first simulation can be calculated as Equations (15) and (16). Then, the ratio of the deformation velocities of the upper part of the glass in No. 1 experiment *V_α_*_1_(*t*), No. 2 experiment *V_β_*_1_(*t*), and No. 3 experiment *V_γ_*_1_(*t*) can be calculated by Equations (17) and (18). The actual simulation results are shown as Equation (19). The error between the model result and the simulation result was about 0.116 for the second simulation, while it was about 0.151 for the third simulation. The error may be caused by the approximation of the contact area in Equation (9). However, Equation (17) plays a certain role in setting the manufacturing parameters.

(15) $$\varepsilon_{\alpha 1}^{\prime }\left(t\right)=\frac{S_{1}}{40\pi V_{\alpha 1}E\tau }e^{-\frac{t}{\tau }}=V_{\alpha 1}\left(t\right),$$

(16) $$V_{\alpha 1}\left(t\right)=\sqrt{S_{1}}\cdot \sqrt{\frac{e^{-\frac{t}{\tau }}}{40\pi E\tau }}=\sqrt{S_{1}}\cdot Y\left(t\right),$$

(17) $$V_{\alpha 1}\left(t\right):V_{\beta 1}\left(t\right):V_{\gamma 1}\left(t\right)=\sqrt{S_{1}}\cdot Y\left(t\right):\sqrt{S_{2}}\cdot Y\left(t\right):\sqrt{S_{3}}\cdot Y\left(t\right)=\sqrt{S_{1}}:\sqrt{S_{2}}:\sqrt{S_{3}},$$

(18) $$V_{\alpha 1}:V_{\beta 1}:V_{\gamma 1}=\sqrt{0.1}:\sqrt{0.2}:\sqrt{0.3}=1:1.414:1.732,$$

(19) $$V_{\alpha 1\_simulation}:V_{\beta 1\_simulation}:V_{\gamma 1\_simulation}=0.025:0.04:0.051=1:1.6:2.04.$$

*V_α_*_1_*simulation*_, *V_β_*_1_*simulation*_, and *V_γ_*_1_*simulation*_ are the deformation velocities of the upper part of the glass in No. 1, No. 2, and No. 3 simulations, respectively. *Y*(*t*) is a function that changes over time.

The ratio of the deformation rates of the glass preforms in the experiments was 0.007:0.012759:0.017852. Thus, the ratio of the rates of the applied force in the experiments was about 0.007^2^:0.012759^2^:0.017852^2^, immediately 1:2.92:5.72. The ratio does not have a linear relationship with the pressing speed. Therefore, the actual press force in the PGM experiments needs to be further studied.

## 5. Conclusions

At present, there is little discussion on the deformation of glass preforms under multi-machining parameters of PGM technology, but more discussion on the properties of the molded lens. This paper investigated the glass preform deformation under multi-machining parameters when fabricating ChG diffractive lens with PGM technology through experiments, FEM simulations, and theoretical analysis. The primary findings are as follows:

1. In different experiments, the ratio of the deformations of the upper half part and the lower half part of the glass preform are approximately a constant when the pressing forces are big enough, but the pressing speeds are set differently. When the pressing force is set linearly over time and the primary pressing force is too small to achieve the setting pressing speed, after a period of time, the deformation speed of the glass preform starts to decrease significantly.
2. FEM can be used to quickly establish the glass preform deformation model in the time when the glass is in a viscoelastic state. The error of the obtained glass preform deformation model is less than 0.16.
3. The established model can be used as a reference to efficiently optimize PGM processing parameters when the designed lens has two different base radii of curvature.

## Figures and Tables

**Table 1:** The coefficients of the designed lens.

Aspherical Surface		Hybrid Aspherical Diffractive Surface	
*c*_1_	0.0368142	*c*_2_	0
*k*_1_	0	*k*_2_	0
*A*_2_	0	*B*_2_	0
*A*_4_	−1.647332 × 10^−5^	*B*_4_	−2.407681 × 10^−5^
*A*_6_	4.918325 × 10^−8^	*B*_6_	2.503151 × 10^−7^
*A*_8_	2.103211 × 10^−10^	*B*_8_	4.310176 × 10^−10^
*A*_10_	−6.120283 × 10^−13^	*B*_10_	−2.210201 × 10^−12^
		*C*_1_	−8.01813 × 10^−4^
		*C*_2_	7.50124 × 10^−7^
-	-	*n*	2.7781

**Table 2:** The processing parameters of the precision glass modelling (PGM) experiments.

No.	Pressing Force (kN)	Pressing Speed (mm/s)	Press Slope (kN/s)	Molds Temperature (°C)
1	4	0.01	0.02	245
2	4	0.2	0.02	245
3	4	2	0.02	245

**Table 3:** The fitting parameters of the displacement of the three PGM experiments.

No.		1		2		3	
Time(s)		0–81	90–1287	0–81	90–1197	0–81	90–1377
Linear	*a*_1_	−0.090844	-	−0.137668	-	−0.265058	-
	*a*_2_	0.007464	-	0.012759	-	0.017852	-
	*R* ^2^	0.995506	-	0.993103	-	0.981373	-
Conic	*b*_1_	−0.059042		−0.149098	-	−0.116147	-
	*b*_2_	0.005536		0.013452	-	0.008827	-
	*b*_3_	0.000021		−0.000008	-	0.000100	-
	*R* ^2^	0.998914		0.993254	-	0.994248	-
Cubic	*d*_1_	−0.030814	0.395903	−0.068143	0.969855	0.037628	1.239269
	*d*_2_	0.002533	0.001795	0.004839	0.000838	−0.007534	0.000581
	*d*_3_	0.000101	−0.000002	0.000219	−7.994 × 10^−7^	0.000532	−5.3402 × 10^−7^
	*d*_4_	−5.8669 × 10^−7^	6.4247 × 10^−10^	−0.000002	3.092 × 10^−10^	−0.000003	1.9127 × 10^−10^
	*R* ^2^	0.999873	0.995207	0.995946	0.990299	0.999151	0.982526

**Table 4:** Thermodynamic characteristics of the glass and mold used in the simulation.

Thermo-Mechanical Property		Glass			Mold
Density (kg/m^3^)		4630			2950
Elastic modulus (MPa)		35,082.2			90,000
Poisson’s ratio		0.233			0.2
Viscoelastic parameters	Term no.	1	2	3	-
	Shear constant (MPa)	14,207	19.279	1.1399 × 10^−12^	-
	Relaxation time (s)	0.044	26.814	9531.53	-

**Table 5:** The fitting parameters of the deformation of the three PGM simulations.

No.		1X		2X		4X	
Parts		Upper	Lower	Upper	Lower	Upper	Lower
Linear	*a*_1_	−0.081899	0.068859	−0.090055	0.063932	−0.130654	0.078368
	*a*_2_	0.010769	0.002312	0.021490	0.004672	0.043978	0.008345
	*R* ^2^	0.998638	0.971262	0.999139	0.982101	0.998576	0.961921

**Table 6:** Personal computer parameters and the simulation operation time.

Properties		Informations
Central Processing Unit		Intel(R) Core(TM) i7-5820K CPU @ 3.30 GHz
Graphic Processing Unit		NVIDIA GeForce GTX 960
Random Access Memory		32.0 GB
Operation time	No. 1	5770 s
	No. 2	8882 s
	No. 3	7117 s

## Data Availability

Data underlying the results presented in this paper are not publicly available at this time but may be obtained from the authors upon reasonable request.

## Nomenclature

*c*_1_, *c*_2_	Curvatures at the center of the aspherical surface and the hybrid aspherical diffractive surface
*k*_1_, *k*_2_	Conic constants of the aspherical surface and the hybrid aspherical diffractive surface
*r*_1_, *r*_2_	Radii of the aspherical surface and the hybrid aspherical diffractive surface
*A*_2_, *A*_4_, *A*_6_, *A*_8_, *A*_10_	Even order coefficients of the aspherical surface
*n*	Coefficient of refraction of the lens
*B*_2_, *B*_4_, *B*_6_, *B*_8_, *B*_10_	Even order coefficients of the aspheric base of the hybrid aspherical diffractive surface
*t*	Time (s)
*y*	Displacement (mm)
*a*_1_, *a*_2_	Coefficients of the linear curve
*b*_1_, *b*_2_, *b*_3_	Coefficients of the conic curve
*d*_1_, *d*_2_, *d*_3_, *d*_4_	Coefficients of the cubic curve
E	Elastic modulus (MPa)
*η*	Viscosity (pa·s)
*τ*	Retardation time (s)
*ε_α_*_1_	Strain of the upper surface in No.1 experiment
*σ_α_*_1_, *σ_α2_*	Normal pressure applied to the upper and lower surface of the glass in No.1 experiment (MPa)
*a*	Rate of change of contact area (mm^2^/s)
*V**_α_*_1*_simulation*_, *V**_β_*_1*_simulation*_, *V**_γ_*_1*_simulation*_	Deformation velocities of the upper part of the glass in No. 1, No. 2, and No. 3 simulations (mm/s)
*V_α_*_1_, *V_α_*_2_	Deformation velocities of the upper and lower part of the glass in No.1 experiment (mm/s)
*S*_1_, *S*_2_, *S*_3_	Actual press slopes of the No.1, No. 2, and No. 3 experiment (N/s)
*R_p_*	Projection radius of the lower contact surface (mm)
*A*	Upper contact surface area (mm^2^)
*σ*_0_	Constant stress (MPa)
*ε*	Strain
*F_n_*	Normal force applied to the lower contact surface (N)
Node_T, Node_B, Node_M	Center nodes of top and bottom surface, and edge intermediate node of the glass preform
*Y*(*t*)	A function that changes over time
*V**_β_*_1_, *V_γ_*_1_	Deformation velocities of the upper part of the glass in No.2 and No.3 experiment (mm/s)
*α*	Calculated constant
Abbreviations	
PGM	Precision glass molding
ChG	Chalcogenide glass
FEM	Finite element method
DLC	Diamond like carbon
